# Lipid metabolism-based machine learning models for predicting large for gestational age in non-diabetic pregnancies

**DOI:** 10.3389/fendo.2026.1758008

**Published:** 2026-05-15

**Authors:** Wanqing Liu, Ya Xu, Chen Mi, Shanling Yan

**Affiliations:** 1 Department of Obstetrics and Gynecology, Deyang People’s Hospital, Deyang, Sichuan, China; 2 Department of Ultrasound, Deyang People’s Hospital, Deyang, Sichuan, China

**Keywords:** large for gestational age, lipid metabolism, machine learning, non-diabetic pregnancy, prediction

## Abstract

**Objective:**

The majority of LGA occurs in normoglycemic pregnancies, yet prediction models integrating lipid metabolism for this population are lacking. This study aimed to develop and validate machine learning models based on lipid metabolic profiles to predict LGA risk in non-diabetic pregnant women.

**Methods:**

We retrospectively analyzed 1,968 singleton pregnancies without diabetes. Lipid profiles were assessed at early pregnancy (11–14 weeks) and mid-pregnancy (20–24 weeks). The dataset was randomly divided into training (*n* = 1,574) and independent test sets (*n* = 394) using stratified sampling. Four machine learning algorithms—logistic regression (LR), random forest (RF), support vector machine (SVM), and extreme gradient boosting (XGBoost)—were developed using nested 5×5 cross-validation to prevent overfitting and ensure unbiased performance estimation. Model performance was evaluated using area under the receiver operating characteristic curve (AUC), and feature importance was assessed using SHAP analysis.

**Results:**

Hyperparameter optimization demonstrated stable convergence across outer folds (coefficient of variation < 1.5% for all models). On the independent test set, XGBoost achieved the highest performance with an AUC of 0.776 (95% CI: 0.730-0.822), significantly outperforming LR (AUC 0.688, *P* < 0.001). SHAP analysis identified pre-pregnancy BMI (mean SHAP value: 1.48), gestational weight gain (0.93), and mid-pregnancy triglycerides (0.73) as the strongest predictors. Lipid metabolism-related parameters collectively accounted for approximately 40% of the model’s predictive capacity.

**Conclusions:**

Machine learning models incorporating lipid metabolic profiles demonstrated superior performance in predicting LGA among non-diabetic pregnancies. This approach enables early risk stratification and may guide personalized prenatal management.

## Introduction

Large for gestational age (LGA), defined as birth weight at or above the 90th percentile for gestational age, is strongly associated with adverse perinatal outcomes (1, 2). Neonates with LGA are at increased risk of delivery complications, including shoulder dystocia, birth trauma, and perinatal asphyxia, with correspondingly higher rates of cesarean delivery. Long-term follow-up studies have demonstrated that individuals born LGA exhibit significantly elevated risks of developing obesity, type 2 diabetes, and cardiovascular disease in adulthood compared to those with normal birth weight (3, 4).

Fetal growth and development are mediated by efficient maternal-fetal nutrient transfer, with lipid metabolism serving as a critical regulator. Throughout pregnancy, maternal plasma concentrations of total cholesterol (TC), triglycerides (TG), and low-density lipoprotein cholesterol (LDL-C) increase progressively across gestation, while high-density lipoprotein cholesterol (HDL-C) exhibits modest elevation peaking in mid-pregnancy. Dysregulated lipid metabolism beyond physiological adaptation may alter transplacental lipid flux, consequently modifying fetal growth trajectories (5–7).

Risk factors for LGA encompass maternal, fetal, and environmental dimensions, including maternal obesity, gestational diabetes mellitus (GDM), prior LGA delivery, multiparity, advanced maternal age, and fetal sex. While GDM represents a well-established risk factor that has been extensively investigated and incorporated into routine screening protocols, approximately 78% of LGA cases occur among normoglycemic pregnant women (8, 9). This suggests that non-glycemic metabolic pathways contribute substantially to LGA pathogenesis in this population; however, predictive studies remain limited, and effective tools for early clinical identification are lacking.

Studies examining the relationship between lipid metabolism and LGA in non-diabetic pregnant women have yielded inconsistent findings. Vrijkotte et al. (10) reported in a large-scale cohort study that each 1 mmol/L increment in maternal TG during early pregnancy was associated with a 48% increased risk of LGA. Similarly, multiple studies have demonstrated an inverse association between HDL-C concentrations and neonatal birth weight (11–13). However, several investigations have failed to confirm these associations, likely attributable to heterogeneity in study population characteristics, sample sizes, gestational timing of lipid assessment, and confounding adjustment strategies (14, 15). Existing studies have predominantly focused on individual lipid parameters in late pregnancy, with insufficient evaluation of the predictive value of lipid alterations during early and mid-gestation. Furthermore, commonly utilized lipid ratio indices, such as TG/HDL-C and apolipoprotein B/apolipoprotein A1 (apoB/apoA1), have not been systematically validated for their utility in LGA prediction.

Traditional predictive models for LGA have been predominantly constructed using logistic regression analysis; however, such approaches exhibit significant limitations when confronted with complex interactions among multiple variables. Machine learning techniques, leveraging their robust data mining capabilities, have demonstrated substantial advantages in the field of perinatal medicine prediction, with multiple studies confirming their superior predictive performance compared to conventional statistical methods (16, 17). Bai et al. (18) reported that machine learning-based predictive models exhibited favorable performance in LGA prediction among women who had received radiotherapy prior to pregnancy. Compared to traditional approaches, machine learning algorithms could automatically identify nonlinear relationships and high-order interactions without requiring pre-specified parametric assumptions. Furthermore, these approaches demonstrate superior performance in handling high-dimensional feature data through automated feature selection and weighting, thereby improving predictive accuracy (19). Nevertheless, investigations of machine learning-based LGA prediction models remain relatively scarce, and predictive models incorporating lipid metabolic profiles specifically for non-diabetic pregnant women have not been reported.

The present study aimed to develop predictive models for LGA based on lipid metabolic parameters during early and mid-pregnancy, systematically evaluating the predictive value of TC, TG, HDL-C, LDL-C, and their derived ratio indices. We employed machine learning algorithms including random forest (RF), support vector machine (SVM), and extreme gradient boosting (XGBoost) to compare their performance in LGA prediction among non-diabetic pregnant women and to benchmark them against traditional logistic regression models, with the ultimate goal of establishing a clinically applicable, lipid metabolism-based risk stratification tool for LGA.

## Materials and methods

### Study design

This retrospective cohort study was approved by the Ethics Committee of Deyang People’s Hospital (2022-04-083-K01), with waiver of informed consent for use of anonymized data. Clinical data were collected from pregnant women who delivered at the obstetric department of Deyang People’s Hospital between January 2020 and December 2023. Demographic characteristics, obstetric history, laboratory test results, and delivery outcomes were extracted from the electronic medical record system. All participants included in the study underwent first prenatal visit during early pregnancy (11–14 weeks of gestation) and completed relevant biochemical assessments during this period. This study was retrospectively registered at the Open Science Framework (OSF; https://osf.io/uhtzv).

### Study participants

Inclusion criteria: (1) aged 18–45 years; (2) singleton pregnancy with delivery at 37–42 weeks of gestation; (3) spontaneous conception; (4) availability of complete clinical data.

Exclusion criteria: (1) pregestational diabetes or GDM; (2) hypertensive disorders of pregnancy (chronic hypertension, gestational hypertension, preeclampsia); (3) pre-pregnancy dyslipidemia; (4) thyroid dysfunction, cardiovascular disease, or other endocrine/metabolic disorders; (5) hepatic, renal, or other major organ dysfunction; (6) chronic viral infections (hepatitis B/C, HIV); (7) use of medications affecting glucose or lipid metabolism during pregnancy; (8) neonatal chromosomal abnormalities, congenital malformations, or inherited metabolic disorders.

**Figure 1:**
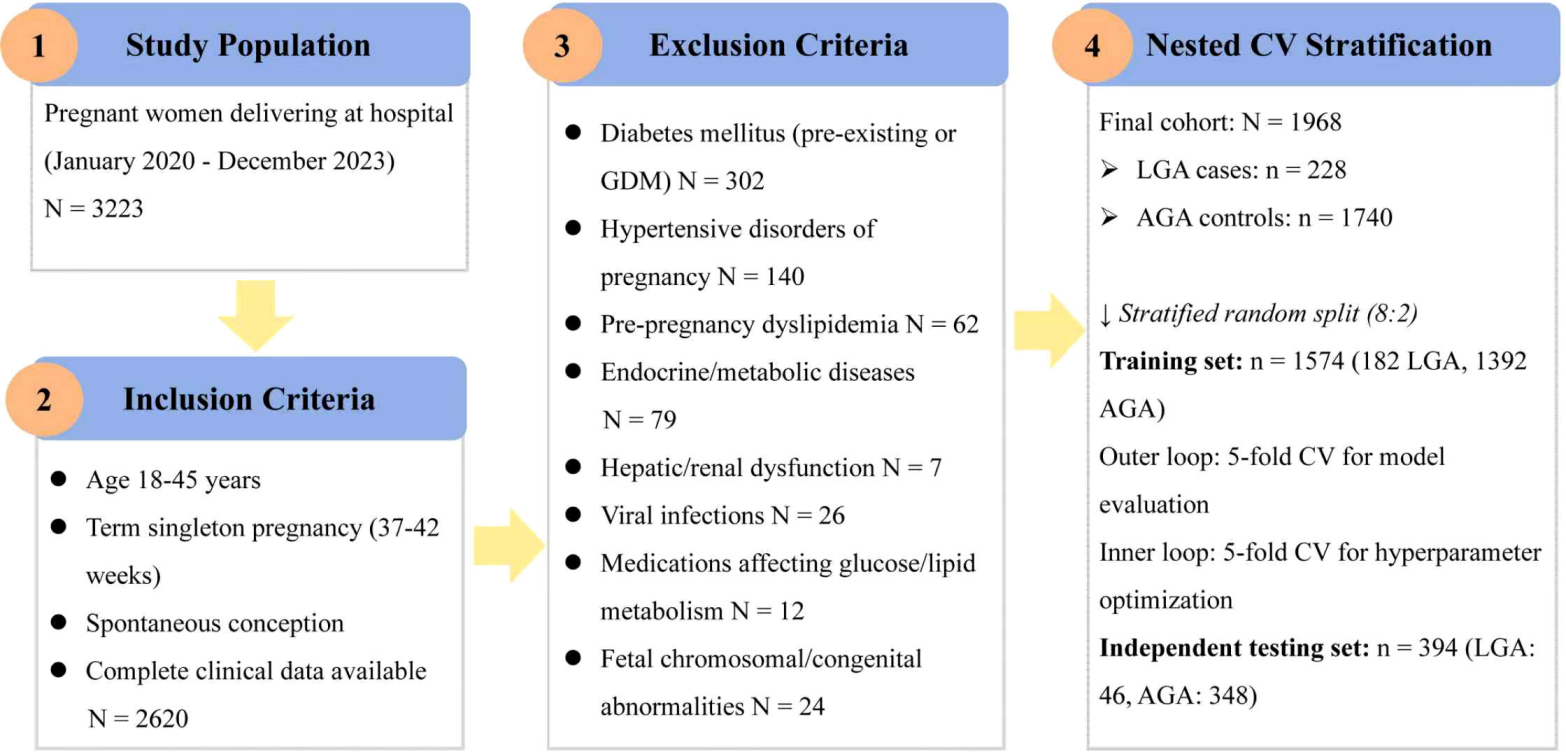
Patient selection flowchart.

According to Chinese neonatal birth weight standards, LGA was defined as birth weight at or above the 90th percentile for gestational age and sex (20). Appropriate for gestational age (AGA) was defined as birth weight between the 10th and 90th percentiles for gestational age and sex. During the study period, 1,968 pregnant women met all inclusion and exclusion criteria, comprising 228 LGA cases and 1,740 AGA cases. The dataset was randomly divided into a training set (*n* = 1,574; 182 LGA cases and 1,392 AGA cases) and an independent test set (*n* = 394; 46 LGA cases and 348 AGA cases) at an 8:2 ratio using stratified random sampling. The study flow diagram is presented in **Figure 1**.

### Data collection

Demographic and clinical data were collected for all participants, including: (1) demographic characteristics: maternal age, gravidity, parity, and education level; (2) medical history: family history of diabetes mellitus in first-degree relatives, smoking and alcohol consumption; (3) anthropometric data: pre-pregnancy weight and height (obtained from medical records or self-report) to calculate pre-pregnancy body mass index (BMI), and mid-pregnancy weight.

Gestational age was calculated based on the last menstrual period and confirmed by early-pregnancy ultrasonography. Pre-pregnancy BMI was calculated as weight (kg) divided by height squared (m^2^), using weight within 3 months before conception. Mid-pregnancy weight was measured at 20–24 weeks of gestation using standardized electronic scales accurate to 0.1 kg (21). Gestational weight gain was calculated as mid-pregnancy weight minus pre-pregnancy weight.

### Laboratory measurements

All participants underwent laboratory assessments during early pregnancy (11–14 weeks of gestation) and mid-pregnancy (20–24 weeks of gestation). After overnight fasting (≥ 12 hours), 5 mL venous blood samples were collected from the antecubital vein in the early morning. Serum was separated by centrifugation and analyzed immediately. Biochemical parameters were measured using an automated biochemical analyzer (Siemens Atellica CH 930, Germany) with matched reagents per manufacturer’s protocol. The following parameters were assessed: fasting plasma glucose (FPG), TC, TG, HDL-C, LDL-C, apoA1 and apoB. All assays met quality control criteria with intra-assay CV ≤ 7.50% and inter-assay CV ≤ 10.00%.

The following lipid ratio indices were calculated: LDL-C/HDL-C, TG/HDL-C, TC/HDL-C, and apoB/apoA1. Change in triglycerides (TG change) was calculated as: TG change = TG (mid-pregnancy) − TG (early pregnancy).

### Ultrasonographic examinations

All participants underwent ultrasonographic examinations as part of routine prenatal care. Early-pregnancy ultrasonography (11–14 weeks) confirmed singleton pregnancy, verified gestational age by crown-rump length (CRL) measurement, measured nuchal translucency (NT), and screened for major fetal structural anomalies.

Mid-pregnancy ultrasonography (20–24 weeks) included systematic screening for fetal structural abnormalities and measurement of fetal biometric parameters: biparietal diameter (BPD), head circumference (HC), abdominal circumference (AC), and femur length (FL). All ultrasonographic examinations were performed by certified sonographers with prenatal diagnostic qualifications with obstetric ultrasound qualifications according to standardized protocols.

### Machine learning model

All candidate features were incorporated as model inputs, with continuous variables z-score standardized and categorical variables one-hot encoded. Class imbalance between LGA and AGA samples (LGA: 11.6%) was addressed through algorithm-specific weighting strategies: logistic regression and SVM employed class weights inversely proportional to class frequencies; RF used balanced class weights; and XGBoost was configured with scale_pos_weight adjustment.

Model development and hyperparameter tuning were conducted using nested 5-fold cross-validation on the training set to prevent overfitting and ensure unbiased performance estimation. The outer cross-validation loop partitioned the training data into 5 folds, with one fold held out for validation and the remaining four used for training in each iteration. An inner 5-fold cross-validation was performed within each outer training fold for hyperparameter optimization.

Four prediction models were developed: LR, RF, SVM, and XGBoost. Hyperparameter optimization was performed via grid search within the inner cross-validation framework. LR was tuned for regularization strength and penalty type (L1/L2); RF for number of estimators, maximum depth, and splitting criteria; SVM (radial basis function kernel) for regularization and kernel parameters; and XGBoost for number of estimators, tree depth, learning rate, and subsampling parameters. Optimal configurations are detailed in **Supplementary Table 1**.

The independent test set was held out throughout model development. For each outer fold, optimal hyperparameters were identified via inner cross-validation, a model was fitted to the complete inner training data, and performance was evaluated on the outer validation fold. Mean performance across the 5 outer folds quantified training set generalization. The best-performing algorithm was then retrained on the complete training set and evaluated on the independent test set for final performance assessment.

### Statistical analysis

Statistical analyses were performed using SPSS 26.0 and Python 3.8 with scikit-learn (version 1.0.2), XGBoost (version 1.5.0), and SHAP (version 0.41.0) libraries. Continuous variables were tested for normality and presented as mean ± standard deviation or median (interquartile range), with group comparisons using t-tests or Mann-Whitney U tests as appropriate. Categorical variables were presented as frequencies (percentages) and compared using chi-square or Fisher’s exact tests. Statistical significance was set at *P* < 0.05.

Model performance was evaluated using the area under the receiver operating characteristic curve (AUC), accuracy, sensitivity, specificity, positive predictive value (PPV), negative predictive value (NPV), and F1-score. Pairwise AUC comparisons were performed using DeLong’s test, with p-values adjusted for multiple comparisons using the Benjamini-Hochberg false discovery rate (FDR) procedure. Model calibration was assessed using Brier scores and calibration plots.

Feature importance was derived from tree-based models (RF and XGBoost) using built-in feature importance scores. Model interpretability was further assessed using SHAP (SHapley Additive exPlanations) values to quantify individual feature contributions to predictions. To enhance the translational utility of the final model, a web-based application was developed for individualized LGA risk estimation. The tool is restricted to non-diabetic singleton pregnancies in which GDM has been excluded (**Supplementary Figure 1**).

## Result

### Baseline characteristics

A total of 1,968 non-diabetic pregnant women were included in this study, with comparable baseline characteristics between the training and test sets. The mean maternal age was 29.9 ± 4.0 years, and pre-pregnancy BMI was 21.2 ± 3.2 kg/m^2^. The majority of participants had attained college-level education or higher (70.38%), 13.82% reported a family history of diabetes in first-degree relatives, and the prevalence of smoking and alcohol consumption was below 2%.

In early pregnancy, median TC, TG, HDL-C, and LDL-C were 4.26, 1.10, 1.76, and 2.15 mmol/L, respectively. Lipid ratio indices showed TG/HDL-C of 0.85, LDL-C/HDL-C of 1.64, and TC/HDL-C of 2.68. Median apoA1 and apoB were 1.46 and 0.81 g/L, respectively, with an apoB/apoA1 ratio of 0.55. FPG remained within normal range at 4.74 ± 0.36 mmol/L (**Table 1**).

**Table 1:** Baseline characteristics and early pregnancy biomarkers.

Maternal characteristics	All (*n* = 1968)	Training set (*n* = 1574)	Test set (*n* = 394)	*P*
Maternal age, years	29.9 ± 4.0	30.0 ± 3.9	29.5 ± 4.2	0.089
Pre-pregnancy BMI, kg/m^2^	21.2 ± 3.2	21.3 ± 3.1	21.1 ± 3.5	0.634
Education level (College or higher), n (%)	1385 (70.38)	1102 (70.01)	283 (71.83)	0.508
Gravidity	1 (1,2)	1 (1,2)	2(1,3)	0.912
Parity	0 (0,1)	0 (0,1)	0 (0,1)	0.934
Family history of diabetes, n (%)	272 (13.82)	220 (13.98)	52 (13.20)	0.695
Alcohol, n (%)	13 (0.66)	10 (0.64)	3 (0.76)	0.841
Smoking, n (%)	32 (1.63)	25 (1.59)	7 (1.78)	0.796
Early pregnancy				
FPG, mmol/L	4.74 ± 0.36	4.73 ± 0.37	4.78 ± 0.33	0.097
TG, mmol/L	1.10 (0.78, 1.43)	1.11 (0.77, 1.42)	1.08 (0.81,1.45)	0.743
TC, mmol/L	4.26 (3.79, 4.91)	4.24 (3.77, 4.85)	4.30 (3.86, 5.12)	0.421
HDL-C, mmol/L	1.76 (1.50, 2.05)	1.75 (1.49, 2.03)	1.78 (1.52, 2.11)	0.689
LDL‐C, mmol/L	2.15 (1.88, 2.75)	2.16 (1.86, 2.76)	2.12 (1.94, 2.72)	0.758
apoA1, g/L	1.46 (1.34, 1.61)	1.47 (1.35, 1.62)	1.45 (1.32, 1.59)	0.612
apoB, g/L	0.81 (0.71, 0.93)	0.82 (0.73, 0.94)	0.79 (0.71, 0.92)	0.156
TG/HDL-C	0.85 (0.60, 1.30)	0.84 (0.59, 1.31)	0.86 (0.61, 1.27)	0.823
LDL-C/HDL-C	1.64 (1.21, 2.05)	1.65 (1.24, 2.07)	1.62 (1.13, 1.98)	0.734
TC/HDL-C	2.68 (2.20, 3.09)	2.69 (2.22, 3.08)	2.64 (2.17, 3.10)	0.678
apoB/apoA1	0.55 (0.42, 0.65)	0.56 (0.43, 0.66)	0.52 (0.40, 0.64)	0.234
Ultrasound indicators				
NT, mm	1.90 ± 0.51	1.91 ± 0.52	1.88 ± 0.48	0.567

P values represent comparisons between training and test sets. BMI, body mass index; FPG, fasting plasma glucose; TG, triglyceride; TC, total cholesterol; HDL-C, high-density lipoprotein cholesterol; LDL-C, low-density lipoprotein cholesterol; apo-A1, apolipoprotein A1; apo-B, apolipoprotein B, NT, nuchal translucency.

By mid-pregnancy, gestational weight gain averaged 7.9 ± 2.4 kg. Lipid concentrations increased substantially, with median TC reaching 5.66 mmol/L, TG 2.04 mmol/L, HDL-C 2.15 mmol/L, and LDL-C 3.15 mmol/L. The TG/HDL-C ratio increased to 1.09 and TC/HDL-C to 3.14. Median TG change was 0.85 mmol/L, while FPG decreased slightly to 4.53 ± 0.35 mmol/L. Mid-pregnancy fetal biometry parameters were BPD 51.6 ± 2.9 mm, HC 183.3 ± 6.9 mm, AC 166.6 ± 10.0 mm, and FL 37.9 ± 2.2 mm (**Table 2**).

**Table 2:** Mid-pregnancy biomarkers and perinatal outcomes.

Mid-pregnancy	All (*n* = 1968)	Training set (*n* = 1574)	Test set (*n* = 394)	*P*
Gestational weight gain, kg	7.9 ± 2.4	7.9 ± 2.5	8.0 ± 2.3	0.523
FPG, mmol/L	4.53 ± 0.35	4.51 ± 0.33	4.6 ± 0.42	0.058
TG, mmol/L	2.04 (1.39, 2.52)	2.02 (1.38, 2.50)	2.09 (1.42, 2.56)	0.456
TC, mmol/L	5.66 (4.73, 6.35)	5.65 (4.74, 6.29)	5.68 (4.72, 6.53)	0.723
HDL-C, mmol/L	2.15 (1.86, 2.46)	2.18 (1.86, 2.51)	2.14 (1.83, 2.44)	0.467
LDL‐C, mmol/L	3.15 (2.47, 3.71)	3.14 (2.45, 3.69)	3.16 (2.53, 3.78)	0.831
apoA1, g/L	1.56 (1.41, 1.73)	1.57 (1.42, 1.74)	1.55 (1.40, 1.72)	0.589
apoB, g/L	1.09 (0.96, 1.23)	1.08 (0.96, 1.23)	1.10 (0.97, 1.24)	0.712
TG/HDL-C	1.09 (0.84, 1.43)	1.10 (0.85, 1.45)	1.08 (0.82, 1.40)	0.767
LDL-C/HDL-C	1.87 (1.60, 2.30)	1.86 (1.59, 2.18)	1.88 (1.61, 2.23)	0.645
TC/HDL-C	3.14 (2.75, 3.63)	3.13 (2.74, 3.62)	3.17 (2.79, 3.64)	0.524
apoB/apoA1	0.66 (0.58, 0.77)	0.67 (0.58, 0.77)	0.65 (0.57, 0.76)	0.623
TG change, mmol/L	0.85 (0.42, 1.28)	0.84 (0.41, 1.26)	0.87 (0.44, 1.31)	0.789
Ultrasound indicators				
BPD, mm	51.6 ± 2.9	51.5 ± 3.0	51.9 ± 2.5	0.298
HC, mm	183.3 ± 6.9	183.4 ± 6.9	182.9 ± 6.8	0.614
AC, mm	166.6 ± 10.0	166.8 ± 10.1	166.1 ± 9.7	0.578
FL, mm	37.9 ± 2.2	37.9 ± 2.3	38.0 ± 2.1	0.812

P values represent comparisons between training and test sets. FPG, fasting plasma glucose; TG, triglyceride; TC, total cholesterol; HDL-C, high-density lipoprotein cholesterol; LDL-C, low-density lipoprotein cholesterol; apo-A1, apolipoprotein A1; apo-B, apolipoprotein B; NT, nuchal translucency; BPD, biparietal diameter; HC, head circumference; AC, abdominal circumference; FL, femur length.

### Model development and performance

#### Hyperparameter optimization

**Figure 2:**
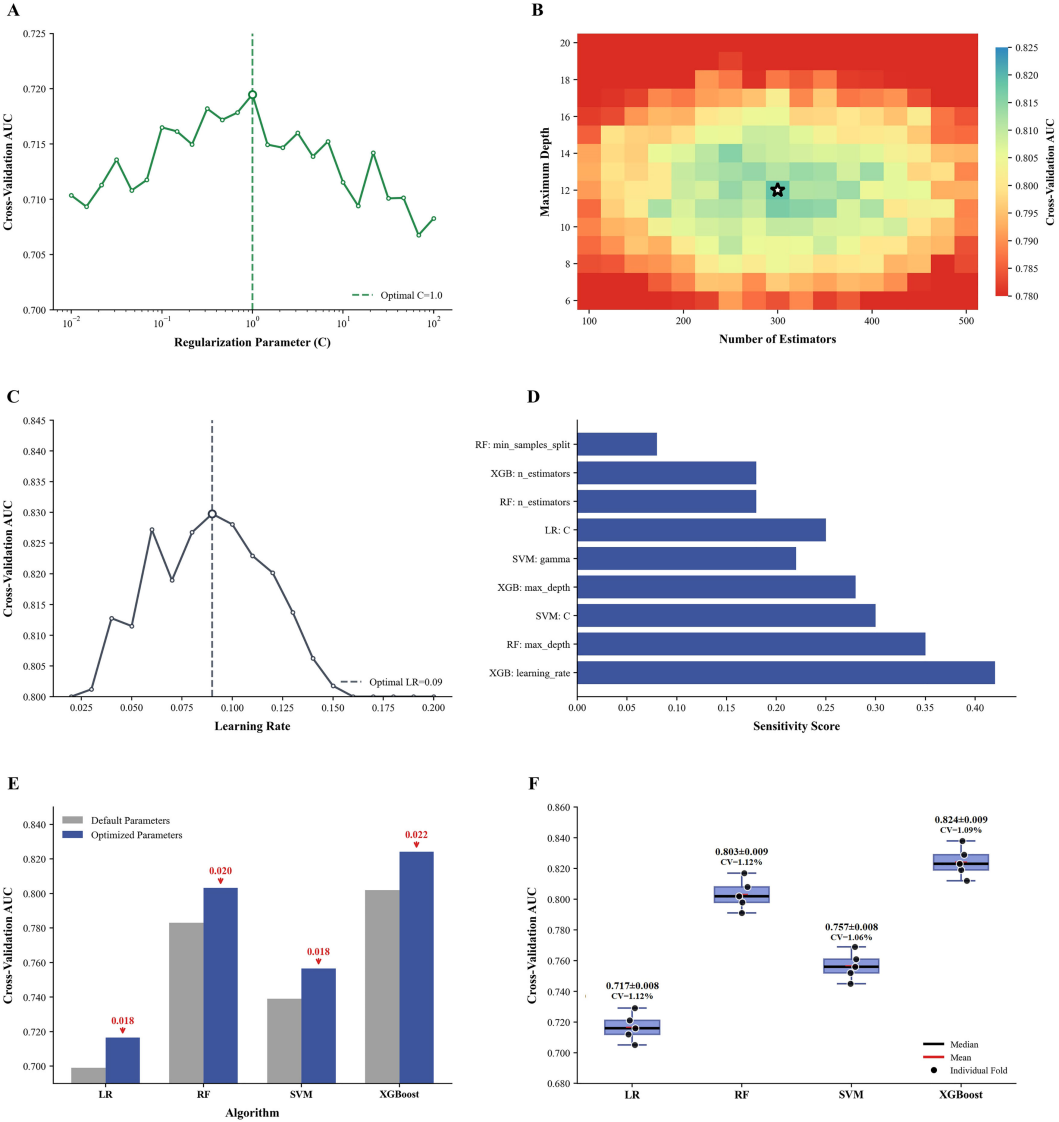
Hyperparameter optimization and cross-validation performance. **(A)** hyperparameter optimization curve for logistic regression showing cross-validation AUC across different regularization parameter **(C)** values. The dashed line indicates the optimal C value that maximizes AUC. **(B)** grid search heatmap for random forest showing cross-validation AUC across combinations of number of estimators and maximum depth. The asterisk marks the optimal parameter combination. **(C)** hyperparameter optimization curve for XGBoost showing cross-validation AUC across different learning rates. The dashed line indicates the optimal learning rate. **(D)** sensitivity scores of key hyperparameters, quantifying their relative impact on model performance. **(E)** performance comparison between default parameters and optimized parameters for each algorithm. Red arrows indicate AUC improvements after optimization. **(F)** distribution of inner cross-validation AUC values across 5 folds for each optimized model during hyperparameter selection. Dots represent individual fold results; black lines show medians; red lines show means. LR, logistic regression; RF, random forest; SVM, support vector machine; XGBoost, extreme gradient boosting; CV, coefficient of variation.

Hyperparameter optimization revealed distinct patterns across algorithms. For logistic regression, the regularization parameter C achieved optimal cross-validation AUC (0.721) at C = 1.0 (**Figure 2A**). Random forest required two-dimensional optimization, with peak performance (AUC = 0.808) observed at 300 trees and maximum depth of 12. The heatmap demonstrated a sharp performance gradient surrounding this optimum, indicating that deviations from these values substantially reduced predictive accuracy (**Figure 2B**). XGBoost learning rate optimization exhibited a unimodal distribution, peaking at 0.09 (AUC = 0.828) with progressive performance degradation at both extremes (**Figure 2C**), resembling the logistic regression pattern but achieving superior AUC values.

Quantitative sensitivity analysis identified XGBoost learning rate as the most influential parameter (sensitivity score: 0.42), followed by random forest maximum depth (0.35) and SVM regularization parameter C (0.30). Logistic regression and SVM kernel parameters exhibited relatively modest sensitivities, ranging from 0.22 to 0.30 (**Figure 2D**). Performance gains from optimization varied across algorithms: XGBoost demonstrated the greatest improvement (ΔAUC = 0.022), followed by RF (0.020), SVM (0.018), and logistic regression (0.018) (**Figure 2E**).

The stability of hyperparameter optimization was assessed through nested cross-validation performance analysis (**Figure 2F**). For each of the 5 outer folds, inner cross-validation identified optimal hyperparameters. The mean AUC during hyperparameter selection (averaged across outer folds) was 0.717 ± 0.008 for logistic regression, 0.757 ± 0.008 for SVM, 0.803 ± 0.009 for RF, and 0.824 ± 0.009 for XGBoost. Coefficients of variation remained below 1.5% for all algorithms, confirming robust parameter selection. These metrics represent the optimal performance observed during the hyperparameter selection phase. These metrics represent the performance achieved during hyperparameter tuning within the inner folds, which are expected to be slightly higher than the subsequent outer fold validation performance due to the use of more training data in the inner loops.

#### Training set generalization performance

**Figure 3:**
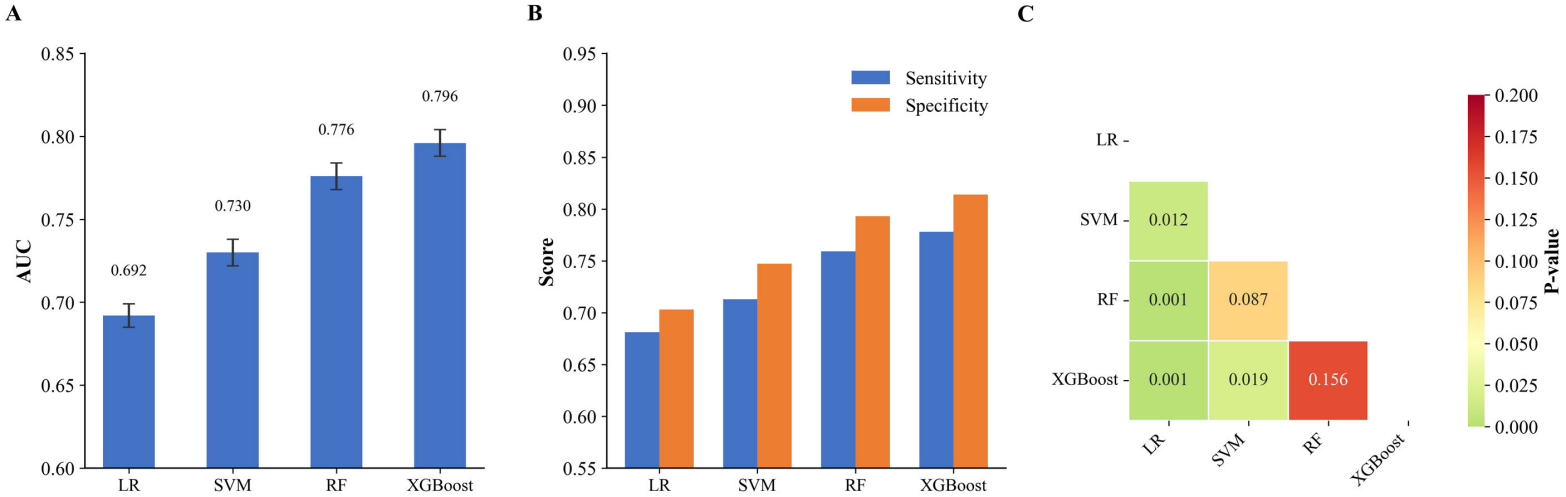
Performance comparison of machine learning models on training set. **(A)** cross-validation AUC values with standard deviation error bars for each algorithm. **(B)** sensitivity (blue bars) and specificity (orange bars) for each algorithm. **(C)** heatmap of DeLong test P-values for pairwise AUC comparisons, with green indicating statistical significance (FDR-adjusted *P* < 0.05). LR, logistic regression; RF, random forest; SVM, support vector machine; XGBoost, extreme gradient boosting; AUC, area under the curve.

Following hyperparameter optimization in the inner loops, model performance was assessed on the independent outer validation folds to estimate true generalization capacity. Model performance was assessed via outer 5-fold cross-validation following hyperparameter optimization. XGBoost achieved the highest mean AUC of 0.796±0.028, followed by RF (0.776±0.032), SVM (0.730±0.028), and logistic regression (0.692±0.028) (**Figure 3A**). All four algorithms exhibited coefficients of variation below 4.5%.

At optimal thresholds, XGBoost yielded sensitivity and specificity values of 0.78 and 0.81, respectively; RF achieved 0.76 and 0.79; SVM demonstrated 0.71 and 0.75; while logistic regression showed 0.68 and 0.70 (**Figure 3B**).

DeLong tests revealed that XGBoost significantly outperformed both logistic regression (P = 0.001) and SVM (P = 0.019), though no significant difference emerged between XGBoost and RF (P = 0.156). RF significantly surpassed logistic regression (P = 0.001) but showed no significant difference from SVM (P = 0.087). SVM demonstrated significantly superior performance over logistic regression (P = 0.012) (**Figure 3C**).

#### Model performance on independent test set

**Figure 4:**
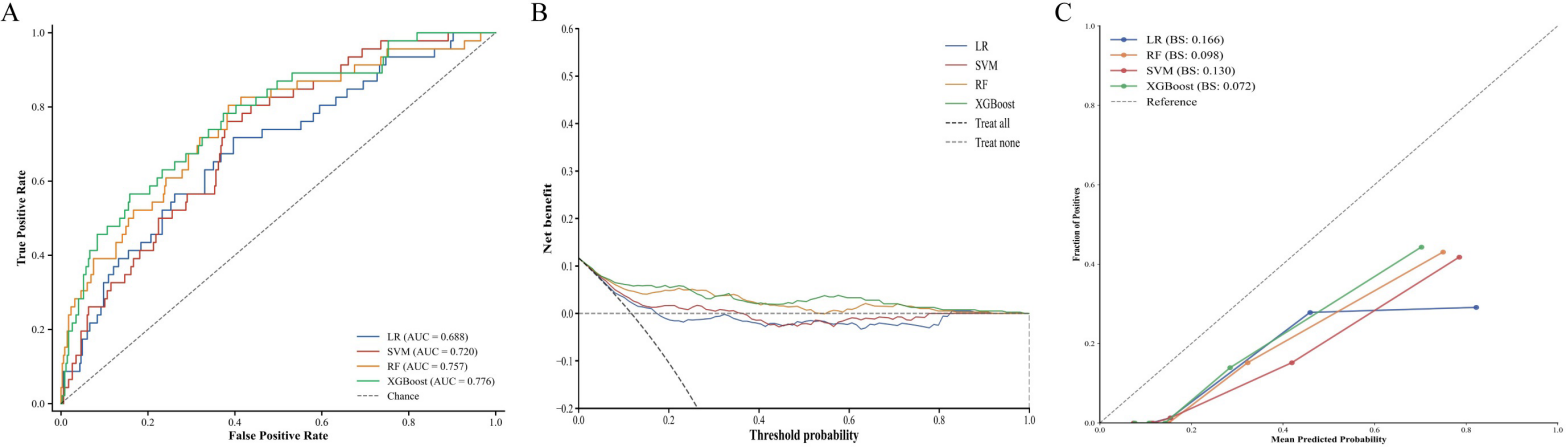
Performance evaluation of machine learning models on the independent test set. **(A)** receiver operating characteristic curves with AUC values for each model; **(B)** decision curve analysis demonstrating net benefit of each model across threshold probabilities. The reference lines represent ‘treat all’ and ‘treat none’ strategies; **(C)** calibration curves with BS quantifying calibration error, where the dashed diagonal line represents perfect calibration. LR, logistic regression; RF, random forest; SVM, support vector machine; XGBoost, extreme gradient boosting; AUC, area under the curve; BS, Brier scores.

Test set performance confirmed robust model generalization. Independent test set evaluation revealed distinct discriminative performance across the four algorithms (**Table 3**, **Figure 4A**). XGBoost demonstrated superior performance with an AUC of 0.776 (95% CI: 0.730-0.822), followed by RF at 0.757 (95% CI: 0.710-0.804), SVM at 0.720 (95% CI: 0.671-0.769), and logistic regression at 0.688 (95% CI: 0.637-0.739). AUC reductions from training to test sets ranged from 0.6% to 2.5%, indicating robust generalization capability.

At optimal classification thresholds, XGBoost achieved sensitivity, specificity, and accuracy of 0.761, 0.793, and 0.789, respectively, with an F1-score of 0.458. RF yielded corresponding values of 0.739, 0.787, 0.782, and 0.442. SVM and logistic regression demonstrated sensitivities of 0.717 and 0.652, respectively, both with specificity of 0.747. Given the low LGA prevalence (11.6%) in the test cohort, all models exhibited high negative predictive values (> 0.94) but relatively modest positive predictive values (0.25-0.33) (**Table 3**).

**Table 3:** Performance comparison of machine learning models on independent test set.

Model	AUC (95% CI)	Accuracy	Sensitivity	Specificity	PPV	NPV	F1-Score
LR	0.688 (0.637-0.739)	0.736	0.652	0.747	0.254	0.942	0.366
RF	0.757 (0.710-0.804)^*^	0.782	0.739	0.787	0.315	0.958	0.442
SVM	0.720 (0.671-0.769)^*^	0.744	0.717	0.747	0.273	0.952	0.395
XGBoost	0.776 (0.730-0.822)^*^	0.789	0.761	0.793	0.327	0.962	0.458

^*^ *P* < 0.05 vs LR. All P-values are FDR-adjusted. LR, logistic regression; RF, random forest; SVM, support vector machine; XGBoost, extreme gradient boosting; AUC, area under the curve.

Decision curve analysis demonstrated positive net benefit for all four models within the threshold probability range of 0.1-0.3, with XGBoost and RF curves positioned favorably relative to SVM and logistic regression (**Figure 4B**). Calibration assessment showed XGBoost achieved the lowest Brier score (0.072), followed by RF (0.098), SVM (0.130), and logistic regression (0.166). Calibration curves revealed good agreement between predicted and observed probabilities for all models except logistic regression, which underestimated risk at higher probability ranges (**Figure 4C**).

### Interpretability analysis

#### Global feature importance

**Figure 5:**
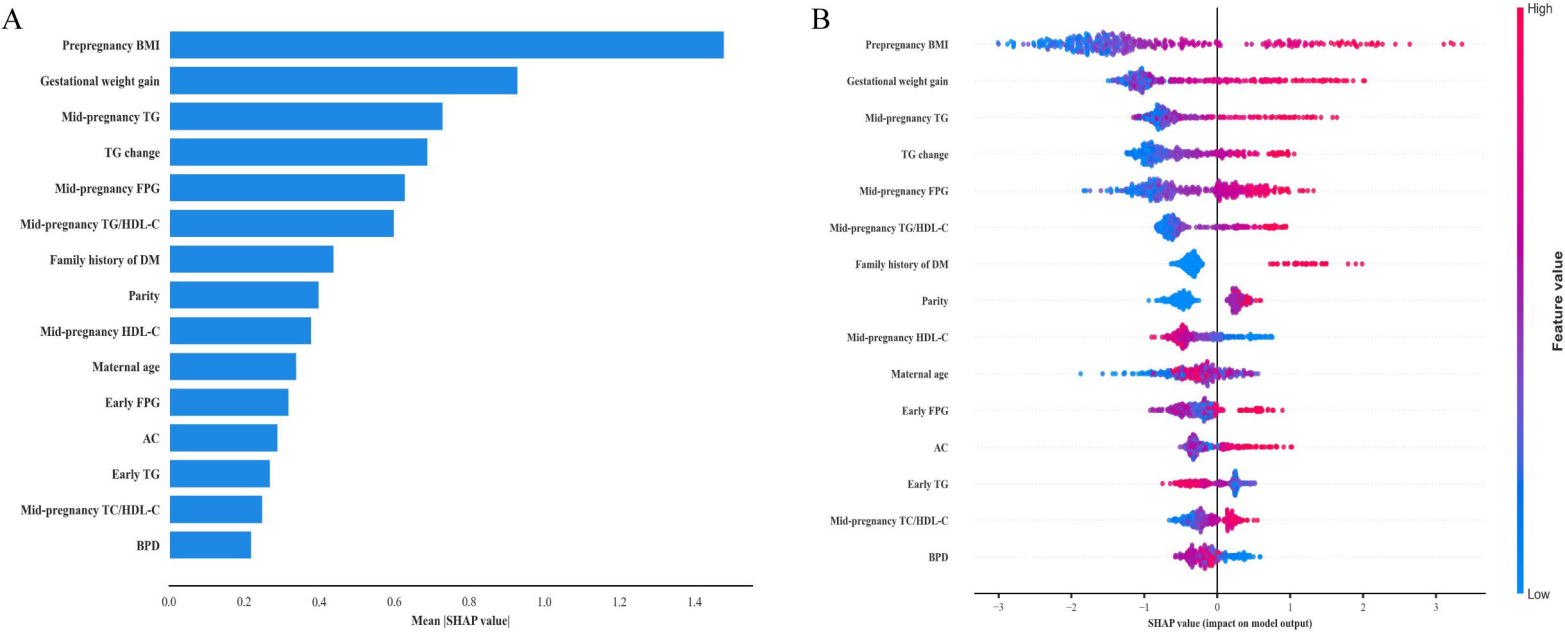
SHAP analysis of feature contributions to large-for-gestational-age prediction. **(A)** feature importance ranking based on mean absolute SHAP values for the top 15 features. **(B)** SHAP summary plot showing distribution of SHAP values and the impact of feature values on model predictions, with color indicating feature magnitude (red: high, blue: low). AC, abdominal circumference; BPD, biparietal diameter; BMI, body mass index; DM, diabetes mellitus; FPG, fasting plasma glucose; HDL-C, high-density lipoprotein cholesterol; SHAP, SHapley Additive exPlanations; TC, total cholesterol; TG, triglyceride.

SHAP analysis identified pre-pregnancy BMI as the most influential predictor (mean absolute SHAP value: 1.48), followed by gestational weight gain (0.93), mid-pregnancy TG (0.73), TG change (0.69), mid-pregnancy FPG (0.63), and mid-pregnancy TG/HDL-C ratio (0.60) (**Figures 5A, B**).

#### Individual feature effects

**Figure 6:**
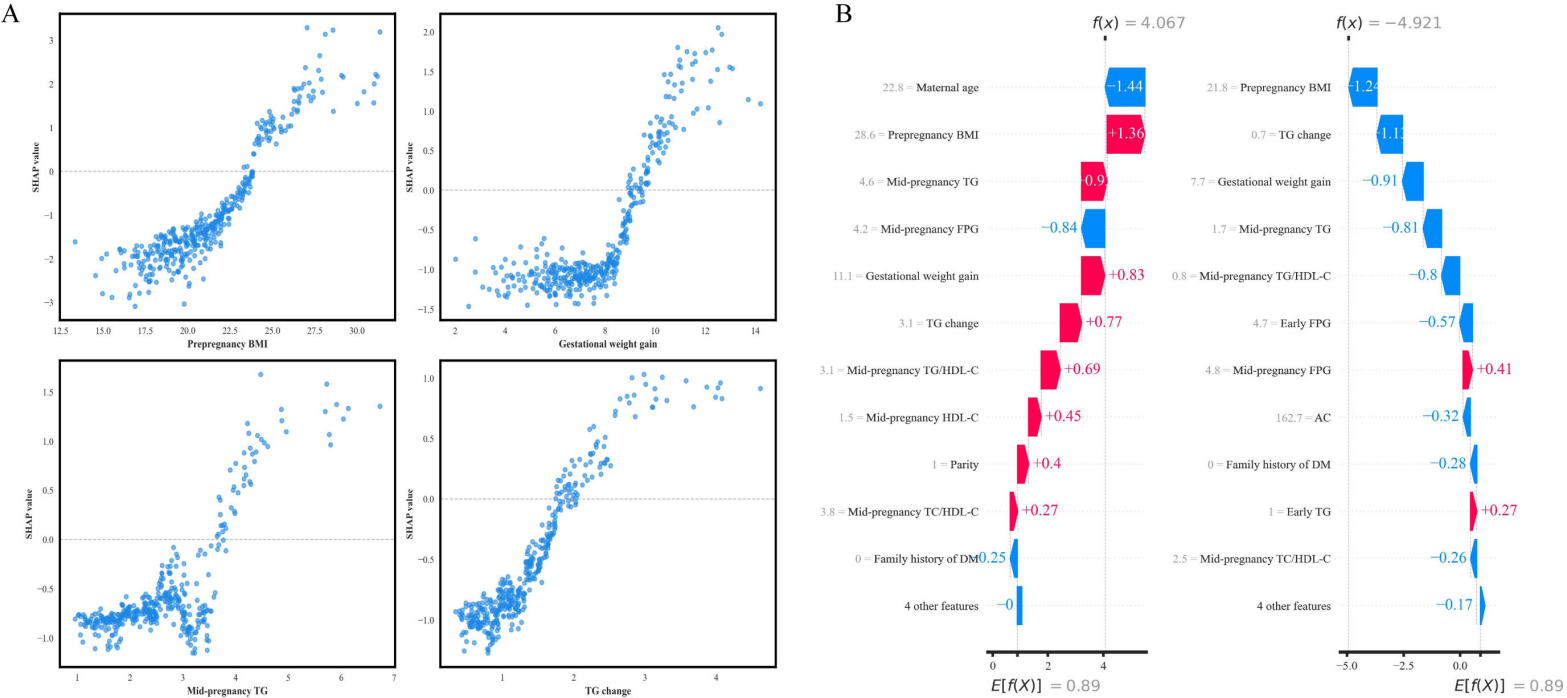
SHAP dependence and waterfall plots illustrating feature impacts on LGA prediction. **(A)** SHAP dependence plots for the four most influential features showing nonlinear relationships between feature values and LGA risk contributions. **(B)** SHAP waterfall plots for representative high-risk (f(x) = 4.067) and low-risk (f(x) = -4.921) cases. Red bars indicate positive contributions; blue bars indicate negative contributions. E[f(X)] = 0.89 represents the average model output. AC, abdominal circumference; BMI, body mass index; DM, diabetes mellitus; FPG, fasting plasma glucose; HDL-C, high-density lipoprotein cholesterol; SHAP, SHapley Additive exPlanations; TC, total cholesterol; TG, triglyceride.

SHAP dependence plots demonstrated non-linear relationships between key features and LGA risk (**Figure 6A**). Pre-pregnancy BMI exhibited a threshold effect, with SHAP values increasing continuously across the observed range and the rate of increase accelerating above approximately 25 kg/m^2^. Gestational weight gain by mid-pregnancy showed progressive risk increases within the observed range. Mid-pregnancy TG and TG change demonstrated positive dose-response relationships with LGA risk.

Waterfall plots illustrated individual-level feature contributions (**Figure 6B**). In a representative high-risk case (*f*(*x*) = 4.067), pre-pregnancy BMI (28.6 kg/m^2^) provided the largest positive contribution (SHAP + 1.36). Additional risk factors included mid-pregnancy TG (4.6 mmol/L, SHAP + 0.9), gestational weight gain (11.1 kg, SHAP + 0.83), TG change (3.1 mmol/L, SHAP + 0.77), and mid-pregnancy TG/HDL-C (3.1, SHAP + 0.69). Conversely, maternal age (22.8 years, SHAP -1.44) and mid-pregnancy FPG (4.2 mmol/L, SHAP -0.84) exerted protective effects.

In a representative low-risk case (*f*(*x*) = -4.921), multiple features contributed to reduced LGA risk, including pre-pregnancy BMI (21.8 kg/m^2^, SHAP -1.24), TG change (0.7 mmol/L, SHAP -1.11), gestational weight gain (7.7 kg, SHAP -0.91), mid-pregnancy TG (1.7 mmol/L, SHAP -0.81), and mid-pregnancy TG/HDL-C (0.8, SHAP -0.8).

## Discussion

This study developed an XGBoost model using data from 1,968 non-diabetic pregnant women to predict LGA risk. The model achieved an AUC of 0.776 on an independent test set, significantly outperforming conventional logistic regression (AUC 0.688). SHAP analysis identified pre-pregnancy BMI, gestational weight gain, and mid-pregnancy triglycerides as the strongest predictors. Lipid metabolism-related parameters collectively contributed approximately 40% of the predictive performance. These findings support the lipid metabolism-based approach to LGA risk stratification in non-diabetic pregnancies, complementing traditional glucose-focused screening strategies.

Most prior studies have examined gestational lipid metabolism and fetal growth in women with GDM or have relied on traditional statistical methods. Machine learning-based prediction models for non-diabetic pregnancies remain limited. Our study addresses this gap by incorporating comprehensive lipid profiles from early to mid-pregnancy with nonlinear modeling approaches. This provides new evidence for LGA risk assessment in non-diabetic pregnant women.

SHAP dependence analysis revealed important dose-response patterns. Pre-pregnancy BMI showed a positive association with LGA risk across the entire range. Risk increased sharply at approximately 25 kg/m^2^, which exceeds the Chinese adult overweight threshold (≥ 24 kg/m^2^) and may represent the transition from adaptive to pathological metabolic adaptation. Gestational weight gain demonstrated continuous risk escalation throughout the observed mid-pregnancy range, suggesting that risk accumulation begins well before total gestational weight gain reaches the IOM-recommended range of 11.5–16 kg for normal-weight women. These findings suggest that individualized risk assessment may be necessary despite adherence to current guidelines.

Prediction models for LGA have traditionally relied on logistic regression. However, these methods show clear limitations when addressing complex interactions among multiple variables. Machine learning techniques have gained traction in perinatal medicine prediction, yet results remain inconsistent. Kuhle et al. (22) applied machine learning to predict abnormal fetal growth in a large cohort of 30,705 pregnancies. The models achieved only modest performance (AUC 0.60-0.73) and failed to demonstrate superiority over logistic regression. The limited predictor set may have prevented the models from leveraging the full potential of machine learning algorithms. Wang et al. (23) collected third-trimester data from 1,285 pregnancies and developed a random forest model incorporating GDM subtypes and glucose-lipid metabolic parameters. Their model showed strong predictive ability (AUC 0.808), suggesting that systematic inclusion of multidimensional metabolic markers enhances model performance. Zhu et al. (24) reinforced this finding by constructing a validated nomogram from multiple clinical and metabolic predictors in GDM pregnancies, confirming that systematic multi-marker integration improves the identification of LGA risk.

Performance variability in machine learning models stems not only from feature selection differences but also from the rigor of validation strategies. Most studies employ simple cross-validation approaches that fail to fully separate hyperparameter optimization from performance evaluation. This can lead to optimistic estimates of model performance (25, 26). To ensure reliable assessment of generalization ability, we implemented nested cross-validation. This method uses nested loops: the inner loop optimizes hyperparameters via grid search, while the outer loop estimates generalization performance on independent data partitions, preventing information leakage (27). Our 5×5 nested architecture ensured strict isolation of test data throughout the modeling process. The optimal hyperparameters showed low variability across the five outer folds (CV < 1.5%), confirming convergence in parameter space exploration. Nested validation is particularly important in perinatal medicine, where sample sizes are often limited relative to the number of predictive variables (28, 29). Within this rigorous validation framework, we systematically incorporated multidimensional lipid metabolic markers from early to mid-pregnancy. This strengthens the biological relevance between predictors and outcomes.

The predictive value of lipid metabolism during pregnancy shows clear gestational age dependency. Early pregnancy represents a transitional phase from baseline to gestational metabolism. Lipid levels during this period are influenced by multiple factors, including pre-pregnancy nutritional status, hormonal fluctuations, and early pregnancy reactions. This results in high inter-individual variability and unstable metabolic patterns (30–32). Starting from 12 weeks of gestation, plasma phospholipids, cholesterol, and triglycerides gradually increase in response to estrogen stimulation and insulin resistance. Placental lipid transporter expression becomes progressively upregulated throughout gestation to support high-throughput lipid transfer. Mid-pregnancy lipid levels (20–24 weeks) reflect the maternal metabolic reserve and adaptive capacity to pregnancy stress. These levels subsequently influence transplacental lipid transport during the period of rapid fetal fat deposition (28–40 weeks) (33, 34). Late pregnancy also demonstrates strong associations between lipid metabolism and fetal growth. Song et al. (35) reported that maternal triglycerides in late pregnancy independently predicted fetal macrosomia in non-diabetic pregnancies, exerting a stronger influence on fetal overgrowth than blood glucose. However, Bozkurt et al. (36) longitudinally tracked lipid trajectories in 222 pregnancies and observed that late-pregnancy TG (>36 weeks) showed associations with LGA prevalence, but measurements at this timepoint offered limited value for clinical management.

We selected early and mid-pregnancy as measurement timepoints. Mid-pregnancy measurements provide both biological relevance and clinical utility. They accurately reflect metabolic imbalance while allowing sufficient time for intervention implementation. Our findings confirmed that mid-pregnancy TG showed substantially greater predictive value than early pregnancy measurements, validating this gestational timing rationale.

SHAP analysis revealed multi-pathway mechanisms underlying LGA development. Pre-pregnancy BMI ranked as the most influential predictor. Its contribution to LGA risk stems not only from pre-existing metabolic imbalance but also from pathological expansion of adipose tissue during pregnancy. The SHAP dependence plot identified a threshold effect at approximately 25 kg/m^2^, above which the contribution to LGA risk increased substantially. Above this threshold, adipocyte dysfunction may lead to ectopic lipid deposition and tissue lipotoxicity, potentially amplifying metabolic dysregulation. Gestational weight gain emerged as the second most important predictor. The SHAP dependence plot showed a continuous upward trend within the 5–12 kg range. Excessive weight gain reflects not only energy surplus but also functional failure of adipose tissue as a lipid buffer. Postprandial free fatty acids increasingly spill into circulation, accelerating maternal triglyceride accumulation.

Mid-pregnancy triglycerides demonstrated substantial predictive importance in the model. As the primary form of maternal energy reserve, elevated TG levels indicate enhanced transplacental lipid transport, promoting fetal fat deposition and overgrowth. Data from the Japan Environment and Children’s Study, encompassing nearly 80,000 pregnancies, showed that LGA risk increased progressively with higher maternal triglyceride levels in both the second and third trimesters (37). SHAP analysis revealed positive dose-response relationships between TG levels and LGA risk. TG change value emerged as an independent predictor, introducing the concept of metabolic trajectory beyond traditional static biochemical markers. TG change provided predictive value independent of absolute TG levels, suggesting that the rate of metabolic adaptation is an important risk determinant (38). Lipid ratio parameters demonstrated predictive value exceeding single parameters. Mid-pregnancy TG/HDL-C ranked sixth in feature importance. As a surrogate marker of insulin resistance, elevated TG/HDL-C reflects multi-level disruption in lipoprotein metabolism. In the pregnancy context, this ratio may identify a subgroup of women who maintain normal glucose tolerance but already exhibit metabolic syndrome phenotype (39).

Traditional LGA prediction studies have emphasized fasting plasma glucose as an important predictor. Maternal glucose concentration shows well-established associations with fetal growth (40, 41). However, in our study, mid-pregnancy FPG demonstrated lower predictive capacity than pre-pregnancy BMI, gestational weight gain, mid-pregnancy TG, and TG change value. This aligns with recent research findings. A machine learning study in Chinese pregnant women found that maternal triglycerides and free fatty acids correlated positively with fetal weight and fat mass, even among GDM women with well-controlled glucose (42). This suggests that lipid metabolic dysregulation may play a critical role in macrosomia development. Herrera et al. (43) further noted that in GDM pregnancies, maternal glucose showed weaker correlations with neonatal birth weight and adiposity than expected. In contrast, lipid parameters including triglycerides and free fatty acids demonstrated significant associations with birth outcomes. These studies indicate that lipid metabolism substantially influences fetal growth, even in pregnancies with glucose abnormalities. This characteristic becomes more pronounced in non-diabetic pregnancies. Our study population maintained glucose levels within the physiological range (mid-pregnancy FPG 4.53 ± 0.35 mmol/L) with minimal inter-individual variation. In contrast, gestational lipid metabolism showed substantial physiological upregulation (mid-pregnancy TG increased approximately 85% from early pregnancy). Lipids serve as the primary substrate for fetal energy supply and fat deposition. Lipid metabolic disruption may more directly impact placental nutrient transport and fetal growth. Beyond glucose, mid-pregnancy ultrasound parameters showed limited predictive value in our study. This finding aligns with previous research, primarily because rapid fetal adipose tissue deposition occurs during late pregnancy (44).

Our findings have important clinical implications for LGA risk assessment in non-GDM pregnancies. Current prenatal care systems emphasize GDM screening but lack systematic prediction tools for the majority of LGA cases occurring in normoglycemic women. Mid-pregnancy lipid testing represents a routine biochemical examination requiring no additional cost. Combined with readily available clinical information such as pre-pregnancy BMI and gestational weight gain, this approach enables early risk identification. High-risk pregnancies can receive more frequent ultrasound monitoring to track fetal growth trajectories dynamically. When necessary, delivery plans can be adjusted under obstetric guidance to reduce complications such as shoulder dystocia. The web-based application described in this study provides individualized LGA probability estimates at the individual patient level, enabling clinicians to quantify LGA risk and visualize the relative contribution of key predictors from routinely available antenatal data.

Several limitations warrant consideration. First, the single-center retrospective design may introduce selection bias, and the ethnically homogeneous population limits generalizability. The higher proportion of participants with college-level education or above (70.4%) reflects the expected patient profile of a tertiary referral hospital and may limit generalizability to primary care settings and rural populations. Second, two-timepoint sampling could not capture continuous metabolic trajectories, and the absence of late-pregnancy measurements may have missed predictive information. Third, unmeasured confounders such as dietary composition, physical activity, and genetic factors may influence the observed associations. Fourth, although all participants underwent routine 75-g OGTT screening, individual 1-hour and 2-hour post-load glucose values were not extracted from medical records and were therefore not included as candidate features. While the modest SHAP contribution of fasting plasma glucose in our model suggests that glycemic variation plays a limited role in this normoglycemic cohort, the potential incremental value of post-load OGTT values and AUC-OGTT cannot be excluded and warrants evaluation in future prospective studies. Fifth, the relatively low PPV of the XGBoost model (0.327) warrants careful clinical interpretation. This largely reflects the inherent mathematical consequence of the low LGA prevalence in our cohort (11.6%), rather than inadequate model discrimination. The model is therefore best positioned as a risk stratification and rule-out tool. The high NPV (0.962) reliably identifies low-risk pregnancies appropriate for standard prenatal care, while a high predicted probability should prompt intensified fetal growth monitoring and individualized counseling rather than being interpreted as an indication for obstetric intervention. Despite rigorous internal validation, external validation in independent cohorts is needed, and the clinical utility of the web-based calculator warrants prospective evaluation. Future multicenter studies should incorporate additional confounders and investigate whether early interventions can reduce LGA incidence.

In conclusion, lipid metabolism-based machine learning models demonstrate good predictive performance for LGA in non-diabetic pregnancies, outperforming conventional logistic regression. These findings support further evaluation of lipid metabolic profiling in prenatal risk assessment and highlight modifiable targets for preventive interventions.

## Data Availability

The original contributions presented in the study are included in the article/**Supplementary Material**. Further inquiries can be directed to the corresponding author.
