# Supplementary material for: Lipid metabolism-based machine learning models for predicting large for gestational age in non-diabetic pregnancies
Source: Front Endocrinol (Lausanne). 2026 May 15;17:1758008. doi: 10.3389/fendo.2026.1758008 (PMC13218891; doi:10.3389/fendo.2026.1758008)
Supplement: Supplementary file 1 [file DataSheet1.docx]

**Supplementary**

Table S1. Optimal hyperparameter configurations for LGA prediction models

| **Algorithm** | **Parameter** | **Optimal Value** | **Search Range** | **Fixed Parameters** |
| --- | --- | --- | --- | --- |
| Logistic Regression | C | 1 | 0.01-100 | penalty='l2' |
|  | penalty | l2 | l1, l2, elasticnet | solver='liblinear' |
|  | class_weight | balanced | None, balanced | max_iter=1000 |
|  |  |  |  | random_state=42 |
| Random Forest | n_estimators | 300 | 100-500 | criterion='gini' |
|  | max_depth | 12 | 6-20 | min_samples_leaf=1 |
|  | min_samples_split | 5 | 2, 5, 10 | max_features='sqrt' |
|  | class_weight | balanced | None, balanced | bootstrap=True |
|  |  |  |  | random_state=42 |
| Support Vector Machine | C | 10 | 0.1-100 | kernel='rbf' |
|  | gamma | 0.1 | 0.001-10 | probability=True |
|  | class_weight | balanced | None, balanced | random_state=42 |
| XGBoost | learning_rate | 0.09 | 0.01-0.2 | objective='binary:logistic' |
|  | n_estimators | 200 | 100-500 | booster='gbtree' |
|  | max_depth | 6 | 3-12 | min_child_weight=1 |
|  | subsample | 0.8 | 0.6-1.0 | colsample_bytree=0.8 |
|  | scale_pos_weight | 7.65 | — | eval_metric='auc' |
|  |  |  |  | random_state=42 |


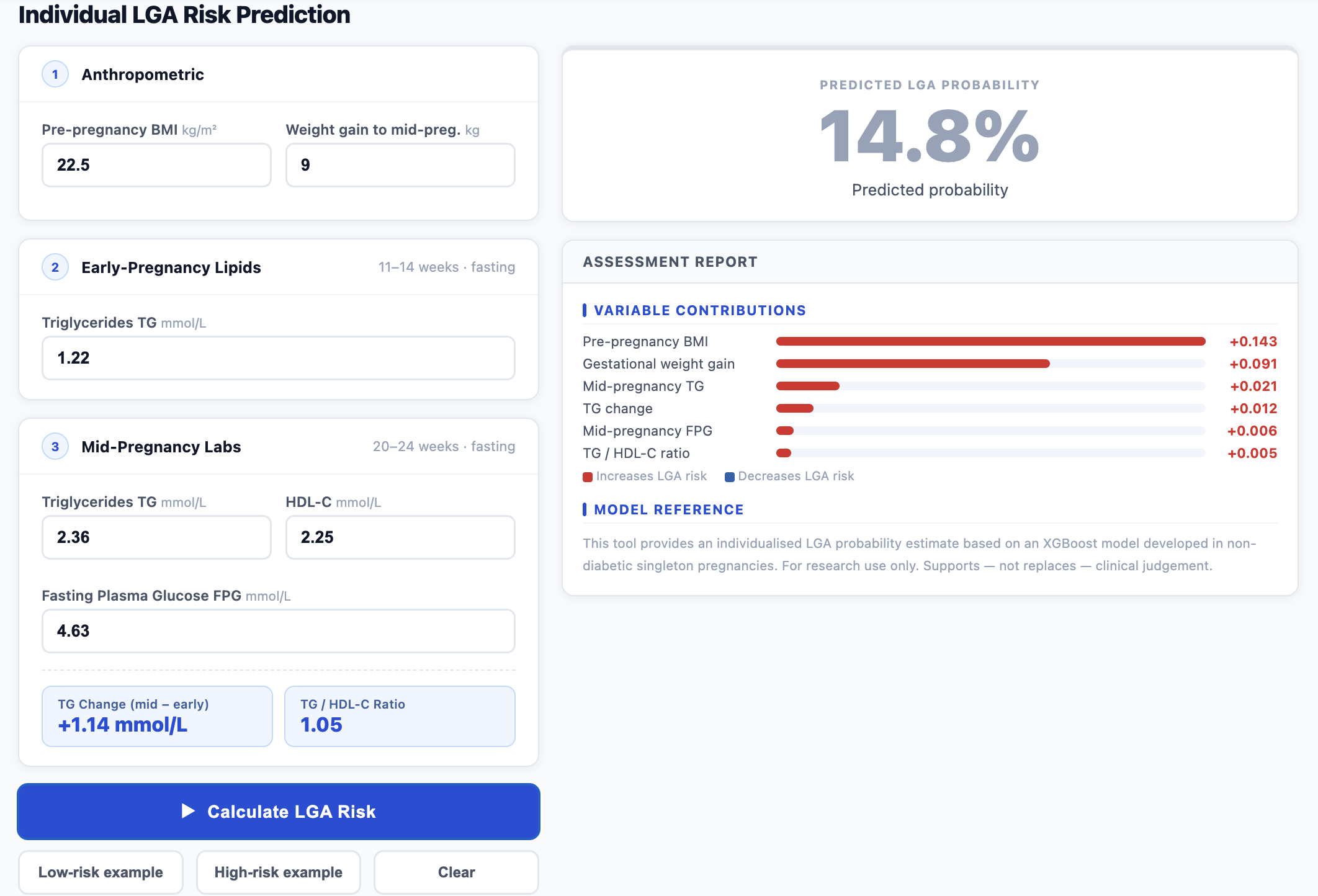


Figure S1. Interactive web-based application for individualised LGA risk estimation
